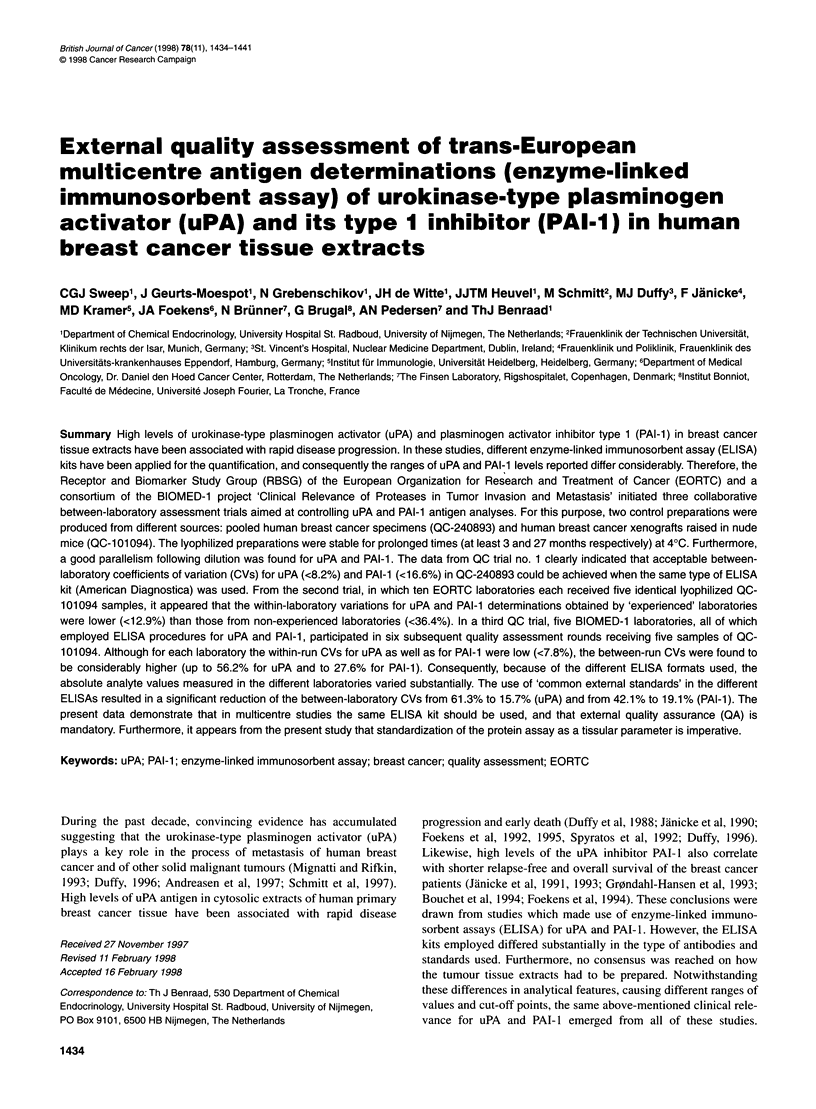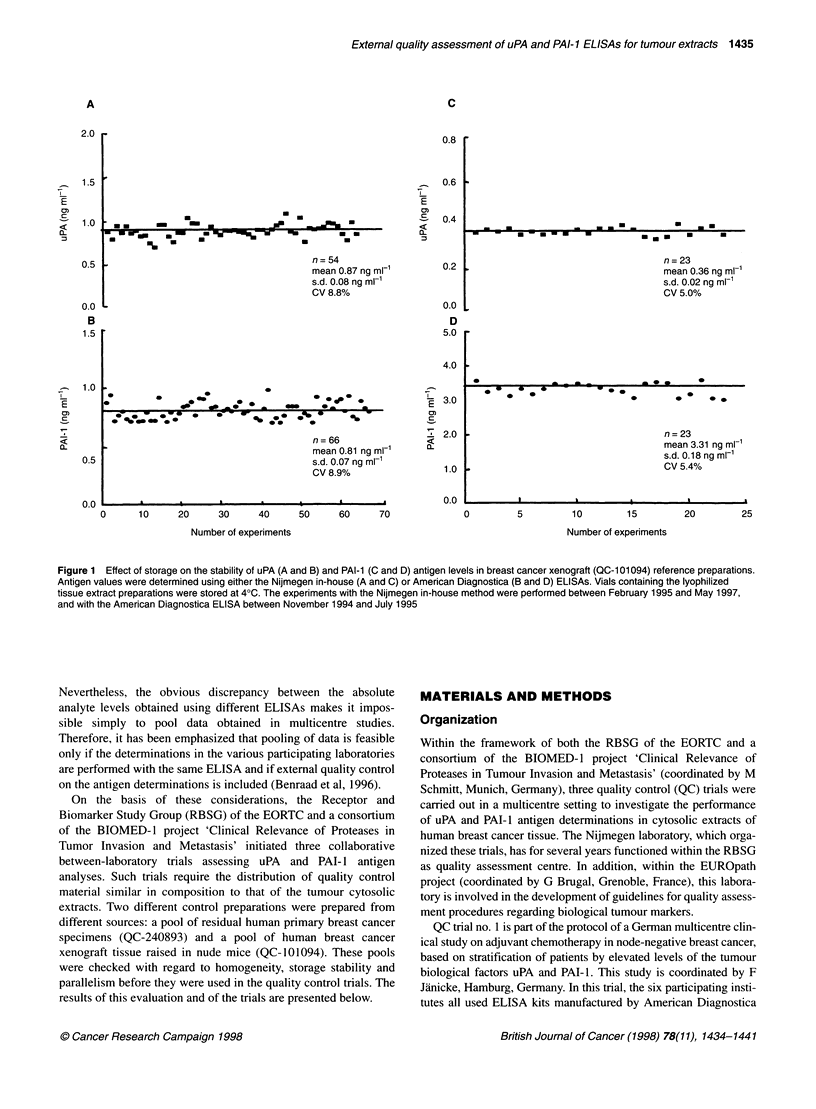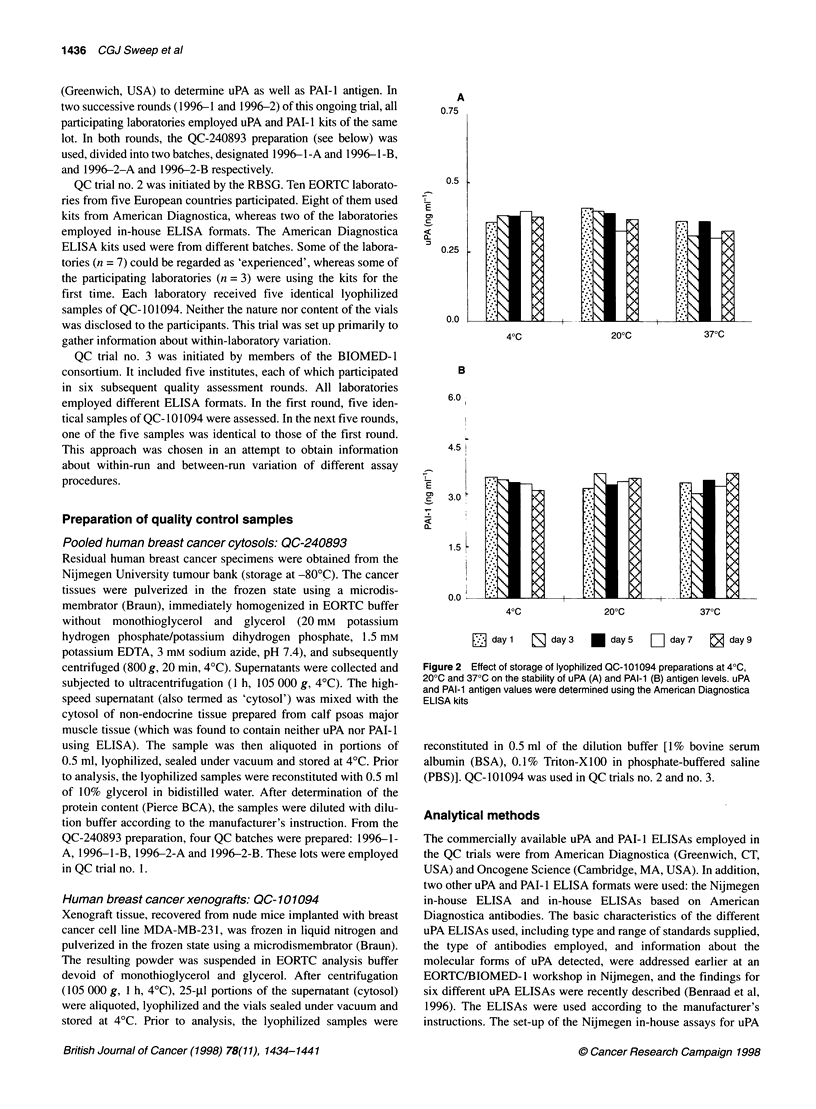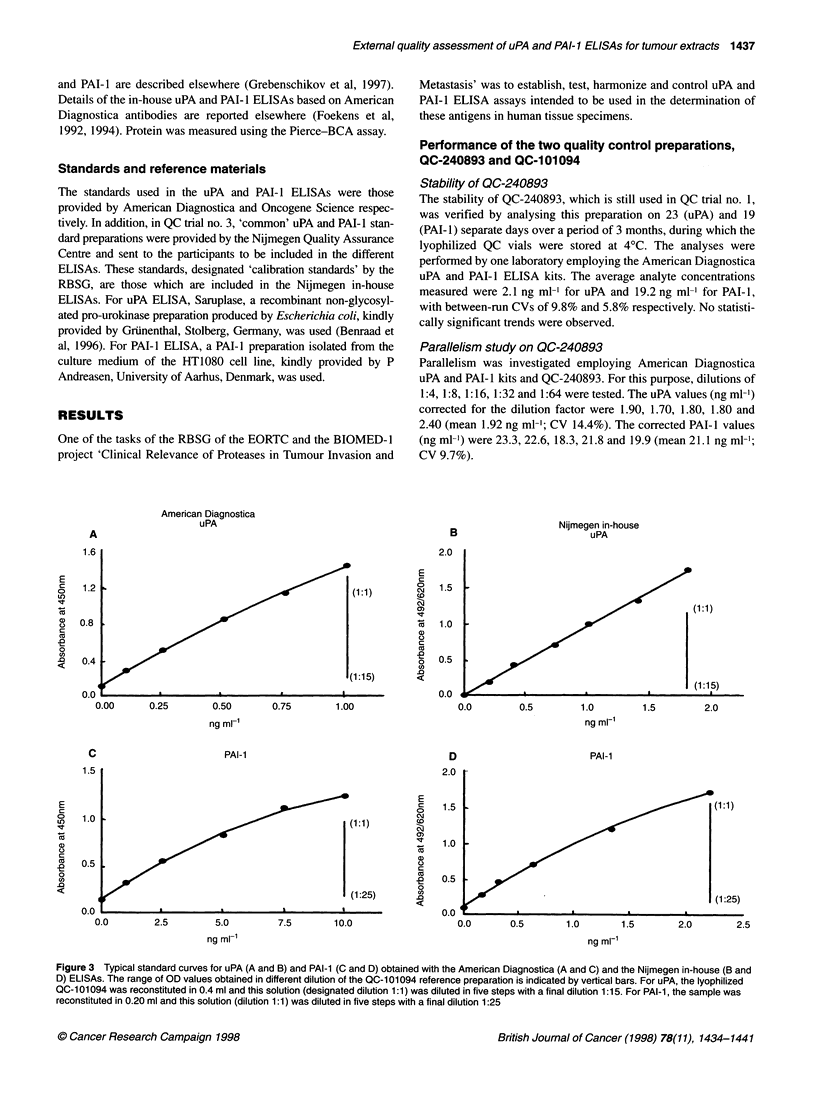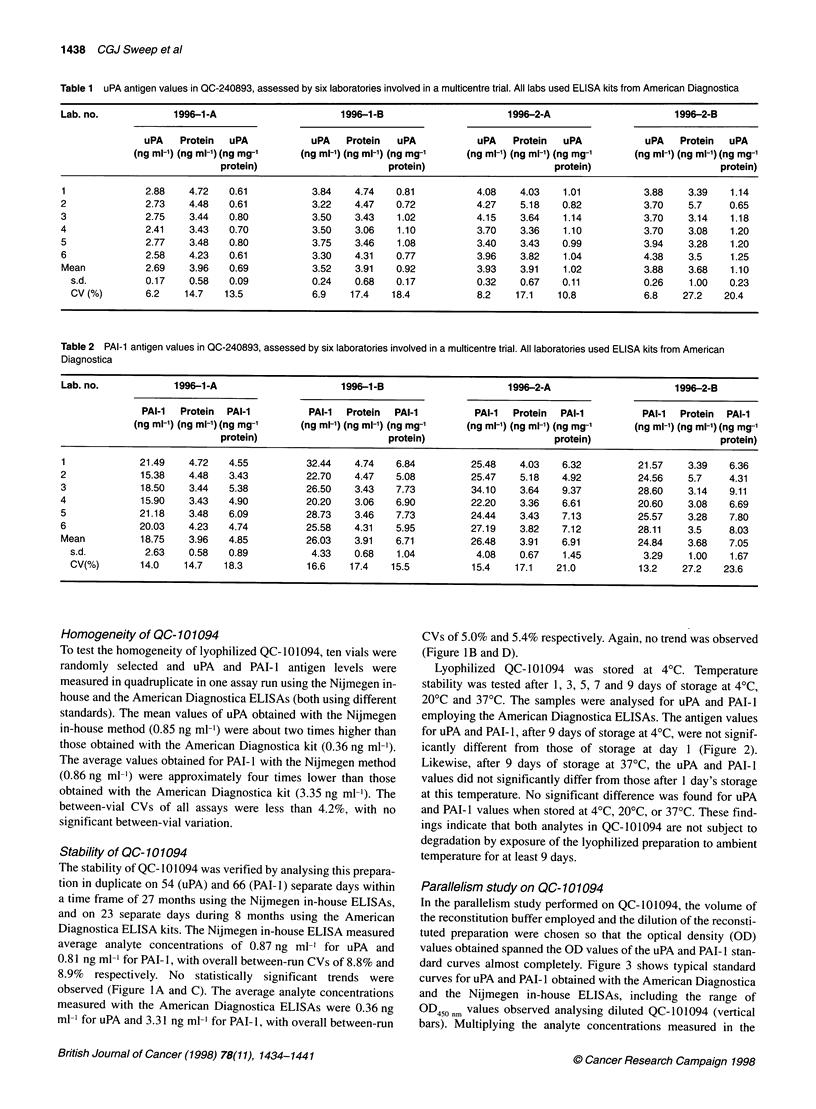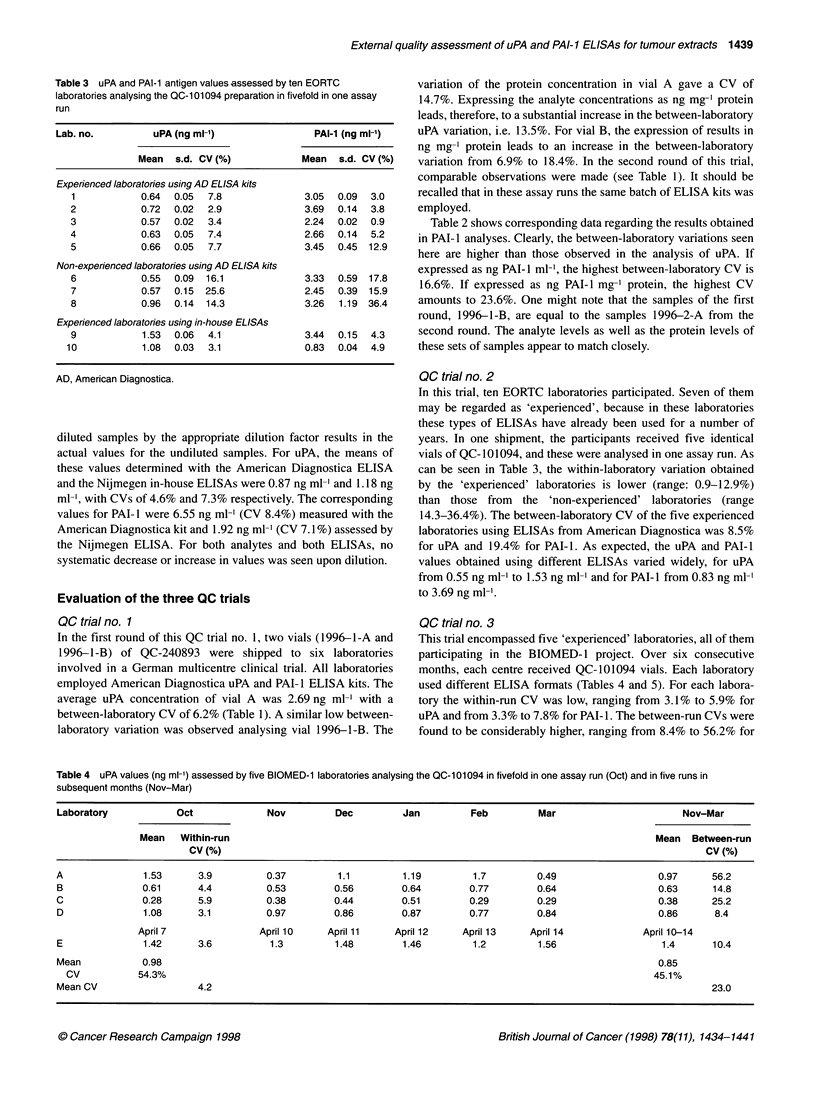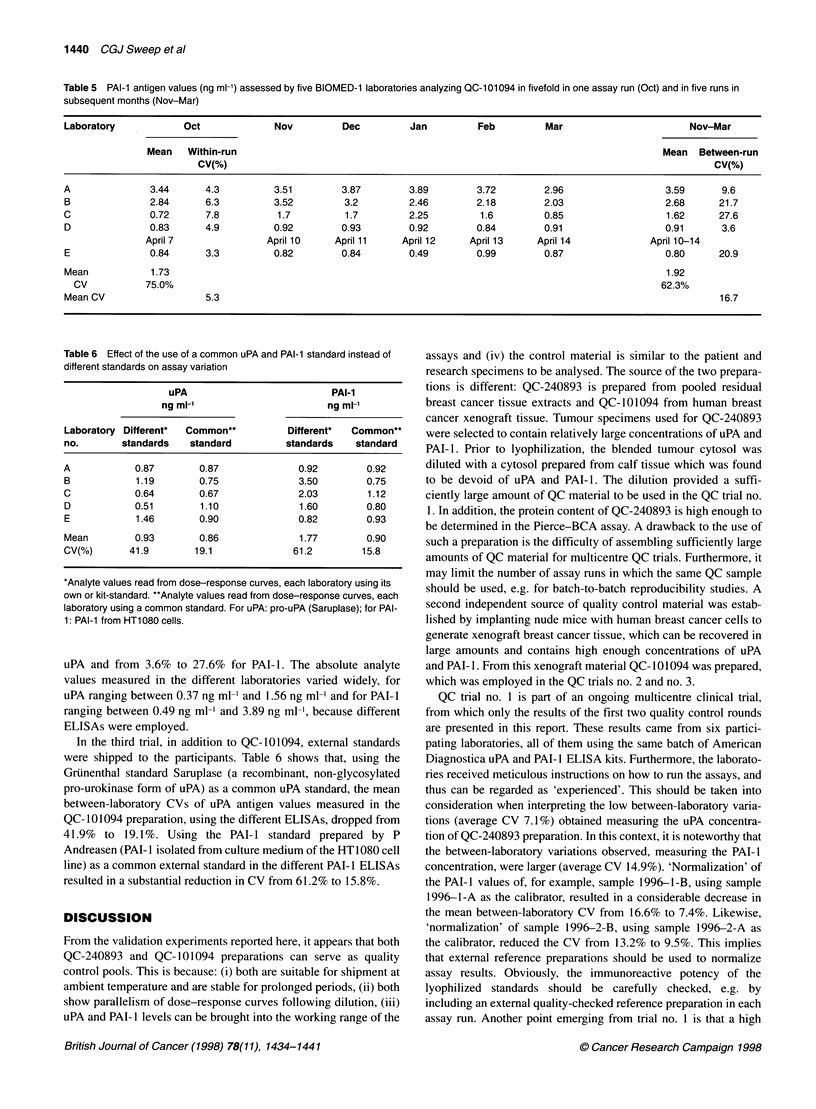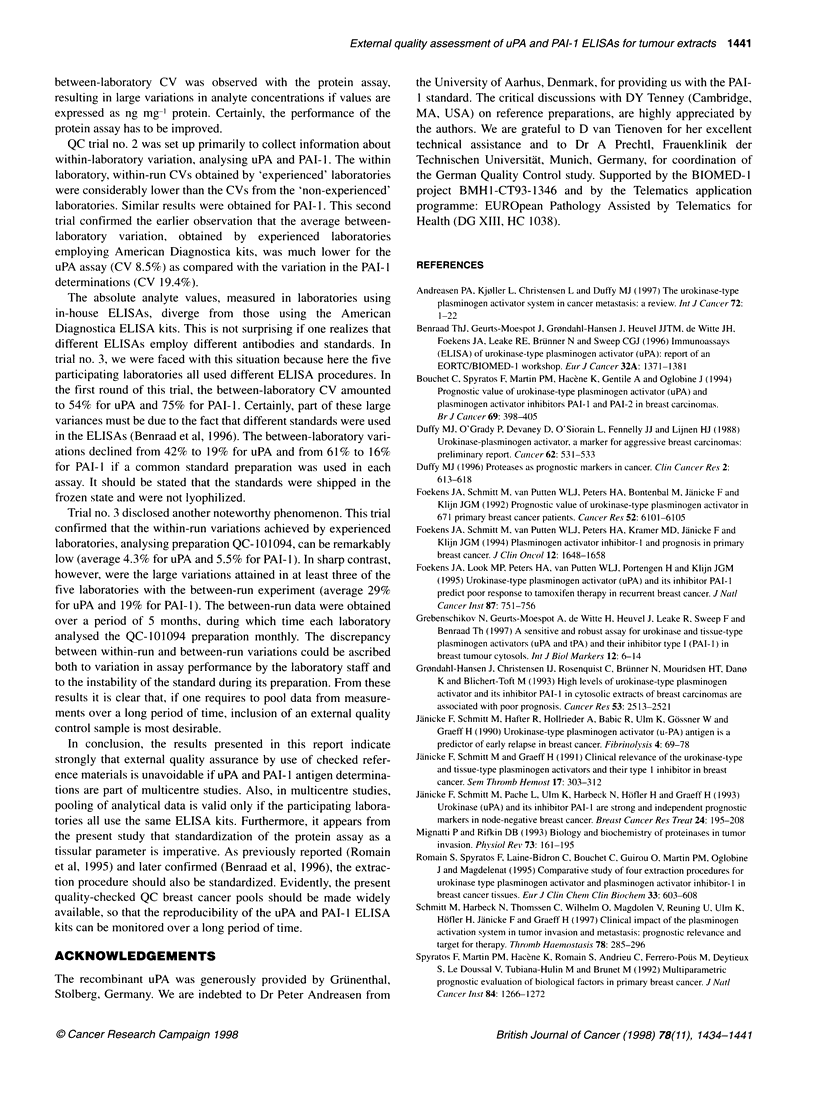# External quality assessment of trans-European multicentre antigen determinations (enzyme-linked immunosorbent assay) of urokinase-type plasminogen activator (uPA) and its type 1 inhibitor (PAI-1) in human breast cancer tissue extracts.

**DOI:** 10.1038/bjc.1998.704

**Published:** 1998-12

**Authors:** C. G. Sweep, J. Geurts-Moespot, N. Grebenschikov, J. H. de Witte, J. J. Heuvel, M. Schmitt, M. J. Duffy, F. Jänicke, M. D. Kramer, J. A. Foekens, N. Brünner, G. Brugal, A. N. Pedersen, T. J. Benraad

**Affiliations:** Department of Chemical Endocrinology, University Hospital St. Radboud, University of Nijmegen, The Netherlands.

## Abstract

High levels of urokinase-type plasminogen activator (uPA) and plasminogen activator inhibitor type 1 (PAI-1) in breast cancer tissue extracts have been associated with rapid disease progression. In these studies, different enzyme-linked immunosorbent assay (ELISA) kits have been applied for the quantification, and consequently the ranges of uPA and PAI-1 levels reported differ considerably. Therefore, the Receptor and Biomarker Study Group (RBSG) of the European Organization for Research and Treatment of Cancer (EORTC) and a consortium of the BIOMED-1 project 'Clinical Relevance of Proteases in Tumor Invasion and Metastasis' initiated three collaborative between-laboratory assessment trials aimed at controlling uPA and PAI-1 antigen analyses. For this purpose, two control preparations were produced from different sources: pooled human breast cancer specimens (QC-240893) and human breast cancer xenografts raised in nude mice (QC-101094). The lyophilized preparations were stable for prolonged times (at least 3 and 27 months respectively) at 4 degrees C. Furthermore, a good parallelism following dilution was found for uPA and PAI-1. The data from QC trial no. 1 clearly indicated that acceptable between-laboratory coefficients of variation (CVs) for uPA (<8.2%) and PAI-1 (<16.6%) in QC-240893 could be achieved when the same type of ELISA kit (American Diagnostica) was used. From the second trial, in which ten EORTC laboratories each received five identical lyophilized QC-101094 samples, it appeared that the within-laboratory variations for uPA and PAI-1 determinations obtained by 'experienced' laboratories were lower (<12.9%) than those from non-experienced laboratories (<36.4%). In a third QC trial, five BIOMED-1 laboratories, all of which employed ELISA procedures for uPA and PAI-1, participated in six subsequent quality assessment rounds receiving five samples of QC-101094. Although for each laboratory the within-run CVs for uPA as well as for PAI-1 were low (<7.8%), the between-run CVs were found to be considerably higher (up to 56.2% for uPA and to 27.6% for PAI-1). Consequently, because of the different ELISA formats used, the absolute analyte values measured in the different laboratories varied substantially. The use of 'common external standards' in the different ELISAs resulted in a significant reduction of the between-laboratory CVs from 61.3% to 15.7% (uPA) and from 42.1% to 19.1% (PAI-1). The present data demonstrate that in multicentre studies the same ELISA kit should be used, and that external quality assurance (QA) is mandatory. Furthermore, it appears from the present study that standardization of the protein assay as a tissular parameter is imperative.


*British Journal of Cancer* (1998) **78**(11), 1434–1441
© 1998 Cancer Research Campaign

### External quality assessment of trans-European multicentre antigen determinations (enzyme-linked immunosorbent assay) of urokinase-type plasminogen activator (uPA) and its type 1 inhibitor (PAI-1) in human breast cancer tissue extracts

**CGJ Sweep[1], J Geurts-Moespot[1], N Grebenschikov[1], JH de Witte[1], JJTM Heuvel[1], M Schmitt[2], MJ Duffy[3], F Jänicke[4], MD Kramer[5], JA Foekens[6], N Brünner[7], G Brugal[8], AN Pedersen[7] and ThJ Benraad[1]**

[1]Department of Chemical Endocrinology, University Hospital St. Radboud, University of Nijmegen, The Netherlands; [2]Frauenklinik der Technischen Universität, Klinikum rechts der Isar, Munich, Germany; [3]St. Vincent's Hospital, Nuclear Medicine Department, Dublin, Ireland; [4]Frauenklinik und Poliklinik, Frauenklinik des Universitäts-krankenhauses Eppendorf, Hamburg, Germany; [5]Institut für Immunologie, Universität Heidelberg, Heidelberg, Germany; [6]Department of Medical Oncology, Dr. Daniel den Hoed Cancer Center, Rotterdam, The Netherlands; [7]The Finsen Laboratory, Rigshospitalet, Copenhagen, Denmark; [8]Institut Bonniot, Faculté de Médecine, Université Joseph Fourier, La Tronche, France

**Summary** High levels of urokinase-type plasminogen activator (uPA) and plasminogen activator inhibitor type 1 (PAI-1) in breast cancer tissue extracts have been associated with rapid disease progression. In these studies, different enzyme-linked immunosorbent assay (ELISA) kits have been applied for the quantification, and consequently the ranges of uPA and PAI-1 levels reported differ considerably. Therefore, the Receptor and Biomarker Study Group (RBSG) of the European Organization for Research and Treatment of Cancer (EORTC) and a consortium of the BIOMED-1 project 'Clinical Relevance of Proteases in Tumor Invasion and Metastasis' initiated three collaborative between-laboratory assessment trials aimed at controlling uPA and PAI-1 antigen analyses. For this purpose, two control preparations were produced from different sources: pooled human breast cancer specimens (QC-240893) and human breast cancer xenografts raised in nude mice (QC-101094). The lyophilized preparations were stable for prolonged times (at least 3 and 27 months respectively) at 4°C. Furthermore, a good parallelism following dilution was found for uPA and PAI-1. The data from QC trial no. 1 clearly indicated that acceptable between-laboratory coefficients of variation (CVs) for uPA (<8.2%) and PAI-1 (<16.6%) in QC-240893 could be achieved when the same type of ELISA kit (American Diagnostica) was used. From the second trial, in which ten EORTC laboratories each received five identical lyophilized QC-101094 samples, it appeared that the within-laboratory variations for uPA and PAI-1 determinations obtained by 'experienced' laboratories were lower (<12.9%) than those from non-experienced laboratories (<36.4%). In a third QC trial, five BIOMED-1 laboratories, all of which employed ELISA procedures for uPA and PAI-1, participated in six subsequent quality assessment rounds receiving five samples of QC-101094. Although for each laboratory the within-run CVs for uPA as well as for PAI-1 were low (<7.8%), the between-run CVs were found to be considerably higher (up to 56.2% for uPA and to 27.6% for PAI-1). Consequently, because of the different ELISA formats used, the absolute analyte values measured in the different laboratories varied substantially. The use of 'common external standards' in the different ELISAs resulted in a significant reduction of the between-laboratory CVs from 61.3% to 15.7% (uPA) and from 42.1% to 19.1% (PAI-1). The present data demonstrate that in multicentre studies the same ELISA kit should be used, and that external quality assurance (QA) is mandatory. Furthermore, it appears from the present study that standardization of the protein assay as a tissular parameter is imperative.

**Keywords:** uPA; PAI-1; enzyme-linked immunosorbent assay; breast cancer; quality assessment; EORTC

During the past decade, convincing evidence has accumulated suggesting that the urokinase-type plasminogen activator (uPA) plays a key role in the process of metastasis of human breast cancer and of other solid malignant tumours (Mignatti and Rifkin, 1993; Duffy, 1996; Andreasen et al, 1997; Schmitt et al, 1997). High levels of uPA antigen in cytosolic extracts of human primary breast cancer tissue have been associated with rapid disease progression and early death (Duffy et al, 1988; Jänicke et al, 1990; Foekens et al, 1992, 1995, Spyratos et al, 1992; Duffy, 1996). Likewise, high levels of the uPA inhibitor PAI-1 also correlate with shorter relapse-free and overall survival of the breast cancer patients (Jänicke et al, 1991, 1993; Grøndahl-Hansen et al, 1993; Bouchet et al, 1994; Foekens et al, 1994). These conclusions were drawn from studies which made use of enzyme-linked immunosorbent assays (ELISA) for uPA and PAI-1. However, the ELISA kits employed differed substantially in the type of antibodies and standards used. Furthermore, no consensus was reached on how the tumour tissue extracts had to be prepared. Notwithstanding these differences in analytical features, causing different ranges of values and cut-off points, the same above-mentioned clinical relevance for uPA and PAI-1 emerged from all of these studies.

*Received 27 November 1997*

*Revised 11 February 1998*

*Accepted 16 February 1998*

*Correspondence to:* Th J Benraad, 530 Department of Chemical Endocrinology, University Hospital St. Radboud, University of Nijmegen, PO Box 9101, 6500 HB Nijmegen, The Netherlands

**Figure 1** Effect of storage on the stability of uPA (A and B) and PAI-1 (C and D) antigen levels in breast cancer xenograft (QC-101094) reference preparations. Antigen values were determined using either the Nijmegen in-house (A and C) or American Diagnostica (B and D) ELISAs. Vials containing the lyophilized tissue extract preparations were stored at 4°C. The experiments with the Nijmegen in-house method were performed between February 1995 and May 1997, and with the American Diagnostica ELISA between November 1994 and July 1995

Nevertheless, the obvious discrepancy between the absolute analyte levels obtained using different ELISAs makes it impossible simply to pool data obtained in multicentre studies. Therefore, it has been emphasized that pooling of data is feasible only if the determinations in the various participating laboratories are performed with the same ELISA and if external quality control on the antigen determinations is included (Benraad et al, 1996).

On the basis of these considerations, the Receptor and Biomarker Study Group (RBSG) of the EORTC and a consortium of the BIOMED-1 project 'Clinical Relevance of Proteases in Tumor Invasion and Metastasis' initiated three collaborative between-laboratory trials assessing uPA and PAI-1 antigen analyses. Such trials require the distribution of quality control material similar in composition to that of the tumour cytosolic extracts. Two different control preparations were prepared from different sources: a pool of residual human primary breast cancer specimens (QC-240893) and a pool of human breast cancer xenograft tissue raised in nude mice (QC-101094). These pools were checked with regard to homogeneity, storage stability and parallelism before they were used in the quality control trials. The results of this evaluation and of the trials are presented below.

### MATERIALS AND METHODS

### Organization

Within the framework of both the RBSG of the EORTC and a consortium of the BIOMED-1 project 'Clinical Relevance of Proteases in Tumour Invasion and Metastasis' (coordinated by M Schmitt, Munich, Germany), three quality control (QC) trials were carried out in a multicentre setting to investigate the performance of uPA and PAI-1 antigen determinations in cytosolic extracts of human breast cancer tissue. The Nijmegen laboratory, which organized these trials, has for several years functioned within the RBSG as quality assessment centre. In addition, within the EUROpath project (coordinated by G Brugal, Grenoble, France), this laboratory is involved in the development of guidelines for quality assessment procedures regarding biological tumour markers.

QC trial no. 1 is part of the protocol of a German multicentre clinical study on adjuvant chemotherapy in node-negative breast cancer, based on stratification of patients by elevated levels of the tumour biological factors uPA and PAI-1. This study is coordinated by F Jänicke, Hamburg, Germany. In this trial, the six participating institutes all used ELISA kits manufactured by American Diagnostica

© Cancer Research Campaign 1998

(Greenwich, USA) to determine uPA as well as PAI-1 antigen. In two successive rounds (1996–1 and 1996–2) of this ongoing trial, all participating laboratories employed uPA and PAI-1 kits of the same lot. In both rounds, the QC-240893 preparation (see below) was used, divided into two batches, designated 1996–1-A and 1996–1-B, and 1996–2-A and 1996–2-B respectively.

QC trial no. 2 was initiated by the RBSG. Ten EORTC laboratories from five European countries participated. Eight of them used kits from American Diagnostica, whereas two of the laboratories employed in-house ELISA formats. The American Diagnostica ELISA kits used were from different batches. Some of the laboratories ($n$ = 7) could be regarded as 'experienced', whereas some of the participating laboratories ($n$ = 3) were using the kits for the first time. Each laboratory received five identical lyophilized samples of QC-101094. Neither the nature nor content of the vials was disclosed to the participants. This trial was set up primarily to gather information about within-laboratory variation.

QC trial no. 3 was initiated by members of the BIOMED-1 consortium. It included five institutes, each of which participated in six subsequent quality assessment rounds. All laboratories employed different ELISA formats. In the first round, five identical samples of QC-101094 were assessed. In the next five rounds, one of the five samples was identical to those of the first round. This approach was chosen in an attempt to obtain information about within-run and between-run variation of different assay procedures.

### Preparation of quality control samples

### *Pooled human breast cancer cytosols: QC-240893*

Residual human breast cancer specimens were obtained from the Nijmegen University tumour bank (storage at –80°C). The cancer tissues were pulverized in the frozen state using a microdismembrator (Braun), immediately homogenized in EORTC buffer without monothioglycerol and glycerol (20 mM potassium hydrogen phosphate/potassium dihydrogen phosphate, 1.5 mM potassium EDTA, 3 mM sodium azide, pH 7.4), and subsequently centrifuged (800 $g$, 20 min, 4°C). Supernatants were collected and subjected to ultracentrifugation (1 h, 105 000 $g$, 4°C). The high-speed supernatant (also termed as 'cytosol') was mixed with the cytosol of non-endocrine tissue prepared from calf psoas major muscle tissue (which was found to contain neither uPA nor PAI-1 using ELISA). The sample was then aliquoted in portions of 0.5 ml, lyophilized, sealed under vacuum and stored at 4°C. Prior to analysis, the lyophilized samples were reconstituted with 0.5 ml of 10% glycerol in bidistilled water. After determination of the protein content (Pierce BCA), the samples were diluted with dilution buffer according to the manufacturer's instruction. From the QC-240893 preparation, four QC batches were prepared: 1996–1-A, 1996–1-B, 1996–2-A and 1996–2-B. These lots were employed in QC trial no. 1.

### *Human breast cancer xenografts: QC-101094*

Xenograft tissue, recovered from nude mice implanted with breast cancer cell line MDA-MB-231, was frozen in liquid nitrogen and pulverized in the frozen state using a microdismembrator (Braun). The resulting powder was suspended in EORTC analysis buffer devoid of monothioglycerol and glycerol. After centrifugation (105 000 $g$, 1 h, 4°C), 25-µl portions of the supernatant (cytosol) were aliquoted, lyophilized and the vials sealed under vacuum and stored at 4°C. Prior to analysis, the lyophilized samples were reconstituted in 0.5 ml of the dilution buffer [1% bovine serum albumin (BSA), 0.1% Triton-X100 in phosphate-buffered saline (PBS)]. QC-101094 was used in QC trials no. 2 and no. 3.

**Figure 2** Effect of storage of lyophilized QC-101094 preparations at 4°C, 20°C and 37°C on the stability of uPA (A) and PAI-1 (B) antigen levels. uPA and PAI-1 antigen values were determined using the American Diagnostica ELISA kits

### Analytical methods

The commercially available uPA and PAI-1 ELISAs employed in the QC trials were from American Diagnostica (Greenwich, CT, USA) and Oncogene Science (Cambridge, MA, USA). In addition, two other uPA and PAI-1 ELISA formats were used: the Nijmegen in-house ELISA and in-house ELISAs based on American Diagnostica antibodies. The basic characteristics of the different uPA ELISAs used, including type and range of standards supplied, the type of antibodies employed, and information about the molecular forms of uPA detected, were addressed earlier at an EORTC/BIOMED-1 workshop in Nijmegen, and the findings for six different uPA ELISAs were recently described (Benraad et al, 1996). The ELISAs were used according to the manufacturer's instructions. The set-up of the Nijmegen in-house assays for uPA

© Cancer Research Campaign 1998

and PAI-1 are described elsewhere (Grebenschikov et al, 1997). Details of the in-house uPA and PAI-1 ELISAs based on American Diagnostica antibodies are reported elsewhere (Foekens et al, 1992, 1994). Protein was measured using the Pierce–BCA assay.

### Standards and reference materials

The standards used in the uPA and PAI-1 ELISAs were those provided by American Diagnostica and Oncogene Science respectively. In addition, in QC trial no. 3, 'common' uPA and PAI-1 standard preparations were provided by the Nijmegen Quality Assurance Centre and sent to the participants to be included in the different ELISAs. These standards, designated 'calibration standards' by the RBSG, are those which are included in the Nijmegen in-house ELISAs. For uPA ELISA, Saruplase, a recombinant non-glycosylated pro-urokinase preparation produced by *Escherichia coli*, kindly provided by Grünenthal, Stolberg, Germany, was used (Benraad et al, 1996). For PAI-1 ELISA, a PAI-1 preparation isolated from the culture medium of the HT1080 cell line, kindly provided by P Andreasen, University of Aarhus, Denmark, was used.

### RESULTS

One of the tasks of the RBSG of the EORTC and the BIOMED-1 project 'Clinical Relevance of Proteases in Tumour Invasion and Metastasis' was to establish, test, harmonize and control uPA and PAI-1 ELISA assays intended to be used in the determination of these antigens in human tissue specimens.

### Performance of the two quality control preparations, QC-240893 and QC-101094

#### *Stability of QC-240893*

The stability of QC-240893, which is still used in QC trial no. 1, was verified by analysing this preparation on 23 (uPA) and 19 (PAI-1) separate days over a period of 3 months, during which the lyophilized QC vials were stored at 4°C. The analyses were performed by one laboratory employing the American Diagnostica uPA and PAI-1 ELISA kits. The average analyte concentrations measured were 2.1 ng $ml^{-1}$ for uPA and 19.2 ng $ml^{-1}$ for PAI-1, with between-run CVs of 9.8% and 5.8% respectively. No statistically significant trends were observed.

#### *Parallelism study on QC-240893*

Parallelism was investigated employing American Diagnostica uPA and PAI-1 kits and QC-240893. For this purpose, dilutions of 1:4, 1:8, 1:16, 1:32 and 1:64 were tested. The uPA values (ng $ml^{-1}$) corrected for the dilution factor were 1.90, 1.70, 1.80, 1.80 and 2.40 (mean 1.92 ng $ml^{-1}$; CV 14.4%). The corrected PAI-1 values (ng $ml^{-1}$) were 23.3, 22.6, 18.3, 21.8 and 19.9 (mean 21.1 ng $ml^{-1}$; CV 9.7%).

**Figure 3** Typical standard curves for uPA (A and B) and PAI-1 (C and D) obtained with the American Diagnostica (A and C) and the Nijmegen in-house (B and D) ELISAs. The range of OD values obtained in different dilution of the QC-101094 reference preparation is indicated by vertical bars. For uPA, the lyophilized QC-101094 was reconstituted in 0.4 ml and this solution (designated dilution 1:1) was diluted in five steps with a final dilution 1:15. For PAI-1, the sample was reconstituted in 0.20 ml and this solution (dilution 1:1) was diluted in five steps with a final dilution 1:25

© Cancer Research Campaign 1998

Table 1 uPA antigen values in QC-240893, assessed by six laboratories involved in a multicentre trial. All labs used ELISA kits from American Diagnostica

| Lab. no. | 1996–1-A | | | 1996–1-B | | | 1996–2-A | | | 1996–2-B | | |
|---|---|---|---|---|---|---|---|---|---|---|---|---|
| | uPA (ng ml$^{-1}$) | Protein (ng ml$^{-1}$) | uPA (ng mg$^{-1}$ protein) | uPA (ng ml$^{-1}$) | Protein (ng ml$^{-1}$) | uPA (ng mg$^{-1}$ protein) | uPA (ng ml$^{-1}$) | Protein (ng ml$^{-1}$) | uPA (ng mg$^{-1}$ protein) | uPA (ng ml$^{-1}$) | Protein (ng ml$^{-1}$) | uPA (ng mg$^{-1}$ protein) |
| 1 | 2.88 | 4.72 | 0.61 | 3.84 | 4.74 | 0.81 | 4.08 | 4.03 | 1.01 | 3.88 | 3.39 | 1.14 |
| 2 | 2.73 | 4.48 | 0.61 | 3.22 | 4.47 | 0.72 | 4.27 | 5.18 | 0.82 | 3.70 | 5.7 | 0.65 |
| 3 | 2.75 | 3.44 | 0.80 | 3.50 | 3.43 | 1.02 | 4.15 | 3.64 | 1.14 | 3.70 | 3.14 | 1.18 |
| 4 | 2.41 | 3.43 | 0.70 | 3.50 | 3.06 | 1.10 | 3.70 | 3.36 | 1.10 | 3.70 | 3.08 | 1.20 |
| 5 | 2.77 | 3.48 | 0.80 | 3.75 | 3.46 | 1.08 | 3.40 | 3.43 | 0.99 | 3.94 | 3.28 | 1.20 |
| 6 | 2.58 | 4.23 | 0.61 | 3.30 | 4.31 | 0.77 | 3.96 | 3.82 | 1.04 | 4.38 | 3.5 | 1.25 |
| Mean | 2.69 | 3.96 | 0.69 | 3.52 | 3.91 | 0.92 | 3.93 | 3.91 | 1.02 | 3.88 | 3.68 | 1.10 |
| s.d. | 0.17 | 0.58 | 0.09 | 0.24 | 0.68 | 0.17 | 0.32 | 0.67 | 0.11 | 0.26 | 1.00 | 0.23 |
| CV (%) | 6.2 | 14.7 | 13.5 | 6.9 | 17.4 | 18.4 | 8.2 | 17.1 | 10.8 | 6.8 | 27.2 | 20.4 |

Table 2 PAI-1 antigen values in QC-240893, assessed by six laboratories involved in a multicentre trial. All laboratories used ELISA kits from American Diagnostica

| Lab. no. | 1996–1-A | | | 1996–1-B | | | 1996–2-A | | | 1996–2-B | | |
|---|---|---|---|---|---|---|---|---|---|---|---|---|
| | PAI-1 (ng ml$^{-1}$) | Protein (ng ml$^{-1}$) | PAI-1 (ng mg$^{-1}$ protein) | PAI-1 (ng ml$^{-1}$) | Protein (ng ml$^{-1}$) | PAI-1 (ng mg$^{-1}$ protein) | PAI-1 (ng ml$^{-1}$) | Protein (ng ml$^{-1}$) | PAI-1 (ng mg$^{-1}$ protein) | PAI-1 (ng ml$^{-1}$) | Protein (ng ml$^{-1}$) | PAI-1 (ng mg$^{-1}$ protein) |
| 1 | 21.49 | 4.72 | 4.55 | 32.44 | 4.74 | 6.84 | 25.48 | 4.03 | 6.32 | 21.57 | 3.39 | 6.36 |
| 2 | 15.38 | 4.48 | 3.43 | 22.70 | 4.47 | 5.08 | 25.47 | 5.18 | 4.92 | 24.56 | 5.7 | 4.31 |
| 3 | 18.50 | 3.44 | 5.38 | 26.50 | 3.43 | 7.73 | 34.10 | 3.64 | 9.37 | 28.60 | 3.14 | 9.11 |
| 4 | 15.90 | 3.43 | 4.90 | 20.20 | 3.06 | 6.90 | 22.20 | 3.36 | 6.61 | 20.60 | 3.08 | 6.69 |
| 5 | 21.18 | 3.48 | 6.09 | 28.73 | 3.46 | 7.73 | 24.44 | 3.43 | 7.13 | 25.57 | 3.28 | 7.80 |
| 6 | 20.03 | 4.23 | 4.74 | 25.58 | 4.31 | 5.95 | 27.19 | 3.82 | 7.12 | 28.11 | 3.5 | 8.03 |
| Mean | 18.75 | 3.96 | 4.85 | 26.03 | 3.91 | 6.71 | 26.48 | 3.91 | 6.91 | 24.84 | 3.68 | 7.05 |
| s.d. | 2.63 | 0.58 | 0.89 | 4.33 | 0.68 | 1.04 | 4.08 | 0.67 | 1.45 | 3.29 | 1.00 | 1.67 |
| CV(%) | 14.0 | 14.7 | 18.3 | 16.6 | 17.4 | 15.5 | 15.4 | 17.1 | 21.0 | 13.2 | 27.2 | 23.6 |

### *Homogeneity of QC-101094*

To test the homogeneity of lyophilized QC-101094, ten vials were randomly selected and uPA and PAI-1 antigen levels were measured in quadruplicate in one assay run using the Nijmegen in-house and the American Diagnostica ELISAs (both using different standards). The mean values of uPA obtained with the Nijmegen in-house method (0.85 ng ml$^{-1}$) were about two times higher than those obtained with the American Diagnostica kit (0.36 ng ml$^{-1}$). The average values obtained for PAI-1 with the Nijmegen method (0.86 ng ml$^{-1}$) were approximately four times lower than those obtained with the American Diagnostica kit (3.35 ng ml$^{-1}$). The between-vial CVs of all assays were less than 4.2%, with no significant between-vial variation.

### *Stability of QC-101094*

The stability of QC-101094 was verified by analysing this preparation in duplicate on 54 (uPA) and 66 (PAI-1) separate days within a time frame of 27 months using the Nijmegen in-house ELISAs, and on 23 separate days during 8 months using the American Diagnostica ELISA kits. The Nijmegen in-house ELISA measured average analyte concentrations of 0.87 ng ml$^{-1}$ for uPA and 0.81 ng ml$^{-1}$ for PAI-1, with overall between-run CVs of 8.8% and 8.9% respectively. No statistically significant trends were observed (Figure 1A and C). The average analyte concentrations measured with the American Diagnostica ELISAs were 0.36 ng ml$^{-1}$ for uPA and 3.31 ng ml$^{-1}$ for PAI-1, with overall between-run CVs of 5.0% and 5.4% respectively. Again, no trend was observed (Figure 1B and D).

Lyophilized QC-101094 was stored at 4°C. Temperature stability was tested after 1, 3, 5, 7 and 9 days of storage at 4°C, 20°C and 37°C. The samples were analysed for uPA and PAI-1 employing the American Diagnostica ELISAs. The antigen values for uPA and PAI-1, after 9 days of storage at 4°C, were not significantly different from those of storage at day 1 (Figure 2). Likewise, after 9 days of storage at 37°C, the uPA and PAI-1 values did not significantly differ from those after 1 day's storage at this temperature. No significant difference was found for uPA and PAI-1 values when stored at 4°C, 20°C, or 37°C. These findings indicate that both analytes in QC-101094 are not subject to degradation by exposure of the lyophilized preparation to ambient temperature for at least 9 days.

### *Parallelism study on QC-101094*

In the parallelism study performed on QC-101094, the volume of the reconstitution buffer employed and the dilution of the reconstituted preparation were chosen so that the optical density (OD) values obtained spanned the OD values of the uPA and PAI-1 standard curves almost completely. Figure 3 shows typical standard curves for uPA and PAI-1 obtained with the American Diagnostica and the Nijmegen in-house ELISAs, including the range of $OD_{450\ nm}$ values observed analysing diluted QC-101094 (vertical bars). Multiplying the analyte concentrations measured in the

© Cancer Research Campaign 1998

**Table 3** uPA and PAI-1 antigen values assessed by ten EORTC laboratories analysing the QC-101094 preparation in fivefold in one assay run

| Lab. no. | uPA (ng ml$^{-1}$) | | | PAI-1 (ng ml$^{-1}$) | | |
|---|---|---|---|---|---|---|
| | Mean | s.d. | CV (%) | Mean | s.d. | CV (%) |
| *Experienced laboratories using AD ELISA kits* | | | | | | |
| 1 | 0.64 | 0.05 | 7.8 | 3.05 | 0.09 | 3.0 |
| 2 | 0.72 | 0.02 | 2.9 | 3.69 | 0.14 | 3.8 |
| 3 | 0.57 | 0.02 | 3.4 | 2.24 | 0.02 | 0.9 |
| 4 | 0.63 | 0.05 | 7.4 | 2.66 | 0.14 | 5.2 |
| 5 | 0.66 | 0.05 | 7.7 | 3.45 | 0.45 | 12.9 |
| *Non-experienced laboratories using AD ELISA kits* | | | | | | |
| 6 | 0.55 | 0.09 | 16.1 | 3.33 | 0.59 | 17.8 |
| 7 | 0.57 | 0.15 | 25.6 | 2.45 | 0.39 | 15.9 |
| 8 | 0.96 | 0.14 | 14.3 | 3.26 | 1.19 | 36.4 |
| *Experienced laboratories using in-house ELISAs* | | | | | | |
| 9 | 1.53 | 0.06 | 4.1 | 3.44 | 0.15 | 4.3 |
| 10 | 1.08 | 0.03 | 3.1 | 0.83 | 0.04 | 4.9 |

AD, American Diagnostica.

diluted samples by the appropriate dilution factor results in the actual values for the undiluted samples. For uPA, the means of these values determined with the American Diagnostica ELISA and the Nijmegen in-house ELISAs were 0.87 ng ml$^{-1}$ and 1.18 ng ml$^{-1}$, with CVs of 4.6% and 7.3% respectively. The corresponding values for PAI-1 were 6.55 ng ml$^{-1}$ (CV 8.4%) measured with the American Diagnostica kit and 1.92 ng ml$^{-1}$ (CV 7.1%) assessed by the Nijmegen ELISA. For both analytes and both ELISAs, no systematic decrease or increase in values was seen upon dilution.

### Evaluation of the three QC trials

### *QC trial no. 1*

In the first round of this QC trial no. 1, two vials (1996–1-A and 1996–1-B) of QC-240893 were shipped to six laboratories involved in a German multicentre clinical trial. All laboratories employed American Diagnostica uPA and PAI-1 ELISA kits. The average uPA concentration of vial A was 2.69 ng ml$^{-1}$ with a between-laboratory CV of 6.2% (Table 1). A similar low between-laboratory variation was observed analysing vial 1996–1-B. The variation of the protein concentration in vial A gave a CV of 14.7%. Expressing the analyte concentrations as ng mg$^{-1}$ protein leads, therefore, to a substantial increase in the between-laboratory uPA variation, i.e. 13.5%. For vial B, the expression of results in ng mg$^{-1}$ protein leads to an increase in the between-laboratory variation from 6.9% to 18.4%. In the second round of this trial, comparable observations were made (see Table 1). It should be recalled that in these assay runs the same batch of ELISA kits was employed.

Table 2 shows corresponding data regarding the results obtained in PAI-1 analyses. Clearly, the between-laboratory variations seen here are higher than those observed in the analysis of uPA. If expressed as ng PAI-1 ml$^{-1}$, the highest between-laboratory CV is 16.6%. If expressed as ng PAI-1 mg$^{-1}$ protein, the highest CV amounts to 23.6%. One might note that the samples of the first round, 1996–1-B, are equal to the samples 1996–2-A from the second round. The analyte levels as well as the protein levels of these sets of samples appear to match closely.

### *QC trial no. 2*

In this trial, ten EORTC laboratories participated. Seven of them may be regarded as 'experienced', because in these laboratories these types of ELISAs have already been used for a number of years. In one shipment, the participants received five identical vials of QC-101094, and these were analysed in one assay run. As can be seen in Table 3, the within-laboratory variation obtained by the 'experienced' laboratories is lower (range: 0.9–12.9%) than those from the 'non-experienced' laboratories (range 14.3–36.4%). The between-laboratory CV of the five experienced laboratories using ELISAs from American Diagnostica was 8.5% for uPA and 19.4% for PAI-1. As expected, the uPA and PAI-1 values obtained using different ELISAs varied widely, for uPA from 0.55 ng ml$^{-1}$ to 1.53 ng ml$^{-1}$ and for PAI-1 from 0.83 ng ml$^{-1}$ to 3.69 ng ml$^{-1}$.

### *QC trial no. 3*

This trial encompassed five 'experienced' laboratories, all of them participating in the BIOMED-1 project. Over six consecutive months, each centre received QC-101094 vials. Each laboratory used different ELISA formats (Tables 4 and 5). For each laboratory the within-run CV was low, ranging from 3.1% to 5.9% for uPA and from 3.3% to 7.8% for PAI-1. The between-run CVs were found to be considerably higher, ranging from 8.4% to 56.2% for

**Table 4** uPA values (ng ml$^{-1}$) assessed by five BIOMED-1 laboratories analysing the QC-101094 in fivefold in one assay run (Oct) and in five runs in subsequent months (Nov–Mar)

| Laboratory | Oct | | Nov | Dec | Jan | Feb | Mar | Nov–Mar | |
|---|---|---|---|---|---|---|---|---|---|
| | Mean | Within-run CV (%) | | | | | | Mean | Between-run CV (%) |
| A | 1.53 | 3.9 | 0.37 | 1.1 | 1.19 | 1.7 | 0.49 | 0.97 | 56.2 |
| B | 0.61 | 4.4 | 0.53 | 0.56 | 0.64 | 0.77 | 0.64 | 0.63 | 14.8 |
| C | 0.28 | 5.9 | 0.38 | 0.44 | 0.51 | 0.29 | 0.29 | 0.38 | 25.2 |
| D | 1.08 | 3.1 | 0.97 | 0.86 | 0.87 | 0.77 | 0.84 | 0.86 | 8.4 |
| | April 7 | | April 10 | April 11 | April 12 | April 13 | April 14 | April 10–14 | |
| E | 1.42 | 3.6 | 1.3 | 1.48 | 1.46 | 1.2 | 1.56 | 1.4 | 10.4 |
| Mean | 0.98 | | | | | | | 0.85 | |
| CV | 54.3% | | | | | | | 45.1% | |
| Mean CV | | 4.2 | | | | | | | 23.0 |

Table 5 PAI-1 antigen values (ng ml$^{-1}$) assessed by five BIOMED-1 laboratories analyzing QC-101094 in fivefold in one assay run (Oct) and in five runs in subsequent months (Nov–Mar)

| Laboratory | Oct | | Nov | Dec | Jan | Feb | Mar | Nov–Mar | |
|---|---|---|---|---|---|---|---|---|---|
| | Mean | Within-run CV(%) | | | | | | Mean | Between-run CV(%) |
| A | 3.44 | 4.3 | 3.51 | 3.87 | 3.89 | 3.72 | 2.96 | 3.59 | 9.6 |
| B | 2.84 | 6.3 | 3.52 | 3.2 | 2.46 | 2.18 | 2.03 | 2.68 | 21.7 |
| C | 0.72 | 7.8 | 1.7 | 1.7 | 2.25 | 1.6 | 0.85 | 1.62 | 27.6 |
| D | 0.83 | 4.9 | 0.92 | 0.93 | 0.92 | 0.84 | 0.91 | 0.91 | 3.6 |
| | April 7 | | April 10 | April 11 | April 12 | April 13 | April 14 | April 10–14 | |
| E | 0.84 | 3.3 | 0.82 | 0.84 | 0.49 | 0.99 | 0.87 | 0.80 | 20.9 |
| Mean | 1.73 | | | | | | | 1.92 | |
| CV | 75.0% | | | | | | | 62.3% | |
| Mean CV | | 5.3 | | | | | | | 16.7 |

Table 6 Effect of the use of a common uPA and PAI-1 standard instead of different standards on assay variation

| | uPA ng ml$^{-1}$ | | PAI-1 ng ml$^{-1}$ | |
|---|---|---|---|---|
| Laboratory no. | Different* standards | Common** standard | Different* standards | Common** standard |
| A | 0.87 | 0.87 | 0.92 | 0.92 |
| B | 1.19 | 0.75 | 3.50 | 0.75 |
| C | 0.64 | 0.67 | 2.03 | 1.12 |
| D | 0.51 | 1.10 | 1.60 | 0.80 |
| E | 1.46 | 0.90 | 0.82 | 0.93 |
| Mean | 0.93 | 0.86 | 1.77 | 0.90 |
| CV(%) | 41.9 | 19.1 | 61.2 | 15.8 |

*Analyte values read from dose–response curves, each laboratory using its own or kit-standard. **Analyte values read from dose–response curves, each laboratory using a common standard. For uPA: pro-uPA (Saruplase); for PAI-1: PAI-1 from HT1080 cells.

uPA and from 3.6% to 27.6% for PAI-1. The absolute analyte values measured in the different laboratories varied widely, for uPA ranging between 0.37 ng ml$^{-1}$ and 1.56 ng ml$^{-1}$ and for PAI-1 ranging between 0.49 ng ml$^{-1}$ and 3.89 ng ml$^{-1}$, because different ELISAs were employed.

In the third trial, in addition to QC-101094, external standards were shipped to the participants. Table 6 shows that, using the Grünenthal standard Saruplase (a recombinant, non-glycosylated pro-urokinase form of uPA) as a common uPA standard, the mean between-laboratory CVs of uPA antigen values measured in the QC-101094 preparation, using the different ELISAs, dropped from 41.9% to 19.1%. Using the PAI-1 standard prepared by P Andreasen (PAI-1 isolated from culture medium of the HT1080 cell line) as a common external standard in the different PAI-1 ELISAs resulted in a substantial reduction in CV from 61.2% to 15.8%.

### DISCUSSION

From the validation experiments reported here, it appears that both QC-240893 and QC-101094 preparations can serve as quality control pools. This is because: (i) both are suitable for shipment at ambient temperature and are stable for prolonged periods, (ii) both show parallelism of dose–response curves following dilution, (iii) uPA and PAI-1 levels can be brought into the working range of the assays and (iv) the control material is similar to the patient and research specimens to be analysed. The source of the two preparations is different: QC-240893 is prepared from pooled residual breast cancer tissue extracts and QC-101094 from human breast cancer xenograft tissue. Tumour specimens used for QC-240893 were selected to contain relatively large concentrations of uPA and PAI-1. Prior to lyophilization, the blended tumour cytosol was diluted with a cytosol prepared from calf tissue which was found to be devoid of uPA and PAI-1. The dilution provided a sufficiently large amount of QC material to be used in the QC trial no. 1. In addition, the protein content of QC-240893 is high enough to be determined in the Pierce–BCA assay. A drawback to the use of such a preparation is the difficulty of assembling sufficiently large amounts of QC material for multicentre QC trials. Furthermore, it may limit the number of assay runs in which the same QC sample should be used, e.g. for batch-to-batch reproducibility studies. A second independent source of quality control material was established by implanting nude mice with human breast cancer cells to generate xenograft breast cancer tissue, which can be recovered in large amounts and contains high enough concentrations of uPA and PAI-1. From this xenograft material QC-101094 was prepared, which was employed in the QC trials no. 2 and no. 3.

QC trial no. 1 is part of an ongoing multicentre clinical trial, from which only the results of the first two quality control rounds are presented in this report. These results came from six participating laboratories, all of them using the same batch of American Diagnostica uPA and PAI-1 ELISA kits. Furthermore, the laboratories received meticulous instructions on how to run the assays, and thus can be regarded as 'experienced'. This should be taken into consideration when interpreting the low between-laboratory variations (average CV 7.1%) obtained measuring the uPA concentration of QC-240893 preparation. In this context, it is noteworthy that the between-laboratory variations observed, measuring the PAI-1 concentration, were larger (average CV 14.9%). 'Normalization' of the PAI-1 values of, for example, sample 1996–1-B, using sample 1996–1-A as the calibrator, resulted in a considerable decrease in the mean between-laboratory CV from 16.6% to 7.4%. Likewise, 'normalization' of sample 1996–2-B, using sample 1996–2-A as the calibrator, reduced the CV from 13.2% to 9.5%. This implies that external reference preparations should be used to normalize assay results. Obviously, the immunoreactive potency of the lyophilized standards should be carefully checked, e.g. by including an external quality-checked reference preparation in each assay run. Another point emerging from trial no. 1 is that a high

© Cancer Research Campaign 1998

between-laboratory CV was observed with the protein assay, resulting in large variations in analyte concentrations if values are expressed as ng $mg^{-1}$ protein. Certainly, the performance of the protein assay has to be improved.

QC trial no. 2 was set up primarily to collect information about within-laboratory variation, analysing uPA and PAI-1. The within laboratory, within-run CVs obtained by 'experienced' laboratories were considerably lower than the CVs from the 'non-experienced' laboratories. Similar results were obtained for PAI-1. This second trial confirmed the earlier observation that the average between-laboratory variation, obtained by experienced laboratories employing American Diagnostica kits, was much lower for the uPA assay (CV 8.5%) as compared with the variation in the PAI-1 determinations (CV 19.4%).

The absolute analyte values, measured in laboratories using in-house ELISAs, diverge from those using the American Diagnostica ELISA kits. This is not surprising if one realizes that different ELISAs employ different antibodies and standards. In trial no. 3, we were faced with this situation because here the five participating laboratories all used different ELISA procedures. In the first round of this trial, the between-laboratory CV amounted to 54% for uPA and 75% for PAI-1. Certainly, part of these large variances must be due to the fact that different standards were used in the ELISAs (Benraad et al, 1996). The between-laboratory variations declined from 42% to 19% for uPA and from 61% to 16% for PAI-1 if a common standard preparation was used in each assay. It should be stated that the standards were shipped in the frozen state and were not lyophilized.

Trial no. 3 disclosed another noteworthy phenomenon. This trial confirmed that the within-run variations achieved by experienced laboratories, analysing preparation QC-101094, can be remarkably low (average 4.3% for uPA and 5.5% for PAI-1). In sharp contrast, however, were the large variations attained in at least three of the five laboratories with the between-run experiment (average 29% for uPA and 19% for PAI-1). The between-run data were obtained over a period of 5 months, during which time each laboratory analysed the QC-101094 preparation monthly. The discrepancy between within-run and between-run variations could be ascribed both to variation in assay performance by the laboratory staff and to the instability of the standard during its preparation. From these results it is clear that, if one requires to pool data from measurements over a long period of time, inclusion of an external quality control sample is most desirable.

In conclusion, the results presented in this report indicate strongly that external quality assurance by use of checked reference materials is unavoidable if uPA and PAI-1 antigen determinations are part of multicentre studies. Also, in multicentre studies, pooling of analytical data is valid only if the participating laboratories all use the same ELISA kits. Furthermore, it appears from the present study that standardization of the protein assay as a tissular parameter is imperative. As previously reported (Romain et al, 1995) and later confirmed (Benraad et al, 1996), the extraction procedure should also be standardized. Evidently, the present quality-checked QC breast cancer pools should be made widely available, so that the reproducibility of the uPA and PAI-1 ELISA kits can be monitored over a long period of time.

### ACKNOWLEDGEMENTS

The recombinant uPA was generously provided by Grünenthal, Stolberg, Germany. We are indebted to Dr Peter Andreasen from the University of Aarhus, Denmark, for providing us with the PAI-1 standard. The critical discussions with DY Tenney (Cambridge, MA, USA) on reference preparations, are highly appreciated by the authors. We are grateful to D van Tienoven for her excellent technical assistance and to Dr A Prechtl, Frauenklinik der Technischen Universität, Munich, Germany, for coordination of the German Quality Control study. Supported by the BIOMED-1 project BMH1-CT93-1346 and by the Telematics application programme: EUROpean Pathology Assisted by Telematics for Health (DG XIII, HC 1038).

### REFERENCES

Andreasen PA, Kjøller L, Christensen L and Duffy MJ (1997) The urokinase-type plasminogen activator system in cancer metastasis: a review. *Int J Cancer* **72**: 1–22

Benraad ThJ, Geurts-Moespot J, Grøndahl-Hansen J, Heuvel JJTM, de Witte JH, Foekens JA, Leake RE, Brünner N and Sweep CGJ (1996) Immunoassays (ELISA) of urokinase-type plasminogen activator (uPA): report of an EORTC/BIOMED-1 workshop. *Eur J Cancer* **32A**: 1371–1381

Bouchet C, Spyratos F, Martin PM, Hacène K, Gentile A and Oglobine J (1994) Prognostic value of urokinase-type plasminogen activator (uPA) and plasminogen activator inhibitors PAI-1 and PAI-2 in breast carcinomas. *Br J Cancer* **69**: 398–405

Duffy MJ, O'Grady P, Devaney D, O'Siorain L, Fennelly JJ and Lijnen HJ (1988) Urokinase-plasminogen activator, a marker for aggressive breast carcinomas: preliminary report. *Cancer* **62**: 531–533

Duffy MJ (1996) Proteases as prognostic markers in cancer. *Clin Cancer Res* **2**: 613–618

Foekens JA, Schmitt M, van Putten WLJ, Peters HA, Bontenbal M, Jänicke F and Klijn JGM (1992) Prognostic value of urokinase-type plasminogen activator in 671 primary breast cancer patients. *Cancer Res* **52**: 6101–6105

Foekens JA, Schmitt M, van Putten WLJ, Peters HA, Kramer MD, Jänicke F and Klijn JGM (1994) Plasminogen activator inhibitor-1 and prognosis in primary breast cancer. *J Clin Oncol* **12**: 1648–1658

Foekens JA, Look MP, Peters HA, van Putten WLJ, Portengen H and Klijn JGM (1995) Urokinase-type plasminogen activator (uPA) and its inhibitor PAI-1 predict poor response to tamoxifen therapy in recurrent breast cancer. *J Natl Cancer Inst* **87**: 751–756

Grebenschikov N, Geurts-Moespot A, de Witte H, Heuvel J, Leake R, Sweep F and Benraad Th (1997) A sensitive and robust assay for urokinase and tissue-type plasminogen activators (uPA and tPA) and their inhibitor type I (PAI-1) in breast tumour cytosols. *Int J Biol Markers* **12**: 6–14

Grøndahl-Hansen J, Christensen IJ, Rosenquist C, Brünner N, Mouridsen HT, Danø K and Blichert-Toft M (1993) High levels of urokinase-type plasminogen activator and its inhibitor PAI-1 in cytosolic extracts of breast carcinomas are associated with poor prognosis. *Cancer Res* **53**: 2513–2521

Jänicke F, Schmitt M, Hafter R, Hollrieder A, Babic R, Ulm K, Gössner W and Graeff H (1990) Urokinase-type plasminogen activator (u-PA) antigen is a predictor of early relapse in breast cancer. *Fibrinolysis* **4**: 69–78

Jänicke F, Schmitt M and Graeff H (1991) Clinical relevance of the urokinase-type and tissue-type plasminogen activators and their type 1 inhibitor in breast cancer. *Sem Thromb Hemost* **17**: 303–312

Jänicke F, Schmitt M, Pache L, Ulm K, Harbeck N, Höfler H and Graeff H (1993) Urokinase (uPA) and its inhibitor PAI-1 are strong and independent prognostic markers in node-negative breast cancer. *Breast Cancer Res Treat* **24**: 195–208

Mignatti P and Rifkin DB (1993) Biology and biochemistry of proteinases in tumor invasion. *Physiol Rev* **73**: 161–195

Romain S, Spyratos F, Laine-Bidron C, Bouchet C, Guirou O, Martin PM, Oglobine J and Magdelenat (1995) Comparative study of four extraction procedures for urokinase type plasminogen activator and plasminogen activator inhibitor-1 in breast cancer tissues. *Eur J Clin Chem Clin Biochem* **33**: 603–608

Schmitt M, Harbeck N, Thomssen C, Wilhelm O, Magdolen V, Reuning U, Ulm K, Höfler H, Jänicke F and Graeff H (1997) Clinical impact of the plasminogen activation system in tumor invasion and metastasis: prognostic relevance and target for therapy. *Thromb Haemostasis* **78**: 285–296

Spyratos F, Martin PM, Hacène K, Romain S, Andrieu C, Ferrero-Poüs M, Deytieux S, Le Doussal V, Tubiana-Hulin M and Brunet M (1992) Multiparametric prognostic evaluation of biological factors in primary breast cancer. *J Natl Cancer Inst* **84**: 1266–1272

© Cancer Research Campaign 1998